# Caffeine Sensitizes U87-MG Human Glioblastoma Cells to Temozolomide through Mitotic Catastrophe by Impeding G2 Arrest

**DOI:** 10.1155/2018/5364973

**Published:** 2018-06-28

**Authors:** Ning Li, Pingde Zhang, Karrie Mei Yee Kiang, Yin Stephen Cheng, Gilberto Ka Kit Leung

**Affiliations:** Department of Surgery, Li Ka Shing Faculty of Medicine, The University of Hong Kong, Queen Mary Hospital, Hong Kong

## Abstract

Temozolomide (TMZ) is the first-line chemotherapeutic agent in the treatment of glioblastoma multiforme (GBM). Despite its cytotoxic effect, TMZ also induces cell cycle arrest that may lead to the development of chemoresistance and eventual tumor recurrence. Caffeine, a widely consumed neurostimulant, shows anticancer activities and is reported to work synergistically with cisplatin and camptothecin. The present study aimed to investigate the effects and the mechanisms of action of caffeine used in combination with TMZ in U87-MG GBM cells. As anticipated, TMZ caused DNA damage mediated by the ATM/p53/p21 signaling pathway and induced significant G2 delay. Concurrent treatment with caffeine repressed proliferation and lowered clonogenic capacity on MTT and colony formation assays, respectively. Mechanistic study showed that coadministration of caffeine and TMZ suppressed the phosphorylation of ATM and p53 and downregulated p21 expression, thus releasing DNA-damaged cells from G2 arrest into premature mitosis. Cell cycle analysis demonstrated that the proportion of cells arrested in G2 phase decreased when caffeine was administered together with TMZ; at the same time, the amount of cells with micronucleation and multipolar spindle poles increased, indicative of enhanced mitotic cell death. Pretreatment of cells with caffeine further enhanced mitotic catastrophe development in combined treatment and sensitized cells to apoptosis when followed by TMZ alone. In conclusion, our study demonstrated that caffeine enhanced the efficacy of TMZ through mitotic cell death by impeding ATM/p53/p21-mediated G2 arrest.

## 1. Introduction

Glioblastoma multiforme (GBM) is the most common and aggressive form of primary brain tumor, with an annual incidence of two to three per million adults. It accounts for more than half of all primary intracranial tumors [1]. Surgical removal followed by radiation and chemotherapy is the standard treatment [2, 3]. However, the recurrence rate remains high, with a median survival of just over one year, rendering GBM one of the most challenging tumors to manage [4].

Temozolomide (TMZ), an oral alkylating agent, is the first-line chemotherapeutics for GBM patients. Its mechanism of action majorly lies in the alkylation of N-7 or O-6 guanine residues or N-3 adenine residues within DNA which leads to mismatches during subsequent DNA replication and consequentially cycle arrest, autophagy, senescence, and cell death [5]. Cell cycle arrest upon DNA damage is thought to be a double-edged sword, however. On the one hand, transient cell cycle delay allows DNA repair and is considered an essential self-protective process in maintaining cellular homeostasis and preventing tumorigenesis in normal tissues. On the other hand, it offers time for tumor cells to erase alkylated residues, correct mismatched base pairs, and eventually promote tumor cell survival, and thereby contributing to chemoresistance and disease recurrence [6]. In this regard, agents that overcome TMZ-induced cell cycle arrest may potentiate its efficacy in GBM treatment.

Caffeine is a widely consumed neurostimulant found in many food products including coffee, tea, and soft drinks. Caffeine exerts multiple biological effects such as raising blood pressure [7], affecting gastrointestinal motility [8], and increasing basal metabolic rate [9]. Because it readily penetrates the blood-brain barrier, caffeine can influence psychological performance, enhance long-term memory, and decrease the risk of neurodegenerative diseases such as Parkinson's disease [10]. Recently, an inverse association between caffeine intake and the risk of brain tumors was reported by two epidemiological studies from the United States and Europe [11, 12]. Laboratory evidence further showed that caffeine alone had a suppressive effect on the proliferation of human U87-MG glioma cells* in vitro* and tumor growth* in vivo *[13]. Moreover, caffeine reduced the migration of GBM cell lines by impairing focal adhesion complex formation [14]. When used in conjunction with cisplatin or camptothecin, enhanced cytotoxicity against human U251 glioma cells was observed [15]. This synergistic cytotoxicity was attributed to the attenuation of chemotherapy-induced G2 cell cycle delay by caffeine. Against this background, we investigated the effects of TMZ with or without concomitant caffeine on U87-MG human glioma cells* in vitro*. We also studied the priming effect of caffeine with a view to mimic the real-life situation of high daily caffeine intake prior to the administration of TMZ. The hypotheses were (i) caffeine would enhance TMZ cytotoxicity by attenuating TMZ-induced G2 arrest; (ii) pretreatment with caffeine would further enhance this effect.

## 2. Materials and Methods

### 2.1. Cell Culture and Drug Treatment

Human GBM cell line U87-MG (American Type Tissue Collection, Manassas, VA, USA) was cultured in minimum essential medium-alpha (MEM-*α*) supplemented with 10% heat-inactivated foetal bovine serum (FBS), 100 IU/ml penicillin, and 100 *μ*g/ml streptomycin (all from Gibco, Life Technologies, Inc., Carlsbad, CA, USA) in a humidified incubator with 95% air atmosphere and 5% carbon dioxide at 37°C. Caffeine powder was obtained from Sigma, and stock solution was prepared in culture medium at the concentration of 32 mM and kept in 4°C. TMZ was obtained from Schering-Plough (Kenilworth, NJ, USA) and dissolved in dimethyl sulfoxide (DMSO, Sigma-Aldrich, Saint Louis, MO, USA) at the concentration 100 mM, further diluted in culture medium to 10 mM, and stored in -20°C. For treatment, caffeine and TMZ were further diluted in medium to the final concentrations as stated below.

**Figure 1:**
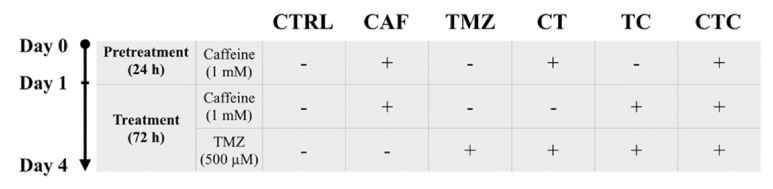
Demonstration of experimental strategies. CTRL: control; CAF: caffeine alone; TMZ: temozolomide alone; CT: temezolomide with caffeine pretreatment; TC: combined treatment of temozolomide and caffeine; and CTC: combined therapy with caffeine pretreatment.

A four-day treatment was divided into two phases: one-day pretreatment and a subsequent three-day treatment of either TMZ alone, caffeine alone, or both (Figure 1). Overall, six arms of cells were set up accordingly. The dosage of caffeine was 1 mM as determined by MTT assay, and the dosage of TMZ we opted was its IC_50_ value defined in U87-MG cells in our previous work [2].

### 2.2. MTT Assay

This was used to define the experimental dosage of caffeine in this study as well as the efficacy of different treatment strategies. Briefly, cells were plated in 96-well microplates at a density of 2000 cells/well overnight for attachment. Caffeine with different concentrations ranging from 0.05 to 10 mM was administered on the second day, and cells were incubated for 96 h. On the fourth day, 10 *μ*l MTT stocking solution (5mg/ml, Sigma-Aldrich) was added on the top of the culture medium, and cells were lysed by adding 100 *μ*l DMSO. Absorbance was acquired at 595 nm using spectrophotometer (MultiSkan FC, Thermo Scientific).

To examine the combined effects of caffeine and TMZ on viability, cells were seeded at a 2 x 10^4^/well in 6-well plates for microscopic imaging or 2 x 10^3^/well in 96-well culture plates for MTT assay, respectively. After full attachment, cells were treated with six different strategies as stated. For microscopic observation, cells in 6-well plates were washed with 1x phosphate buffered saline (PBS) and fixed by 4% paraformaldehyde for image taking. MTT assay was conducted to test the cytotoxicity of different treatment as previously described. Three experiments were performed separately in quadruplicates.

### 2.3. Colony Formation Assay

For longer-term observation, a clonogenic assay was performed. Briefly, 100 cells/well were seeded in 6-well plates and incubated overnight to allow full attachment. On the following four days, different treatment strategies were applied as stated above except that the concentration of TMZ was reduced from 500 *μ*M to 50 *μ*M. All cells were released free on the fifth day and allowed to be maintained in fresh medium for another 10 days. Then cells were fixed with 75% ethanol and stained with crystal violet solution (5g/L, Sigma-Aldrich). Colonies comprising more than fifty cells in each well were counted and analyzed under microscopy.

### 2.4. Cell Cycle Analysis

Cells were harvested and washed with PBS and fixed in 70% ethanol at 4°C overnight. On the following day, cells were washed twice with PBS and resuspended in staining solution containing 20 *μ*g/ml propidium iodide and 100 *μ*g/ml RNase (both from Thermo Fisher Scientific Inc., Waltham, MA) in PBS. After maintaining the reaction in the dark at 37°C for 60 min, cells were washed and resuspended in PBS before analysis on BD FACSCalibur flow cytometry; results were analyzed using FlowJo software.

### 2.5. Immunoblotting

Treated cells were washed with ice-cold PBS twice and collected for homogenization with lysis buffer (Cell Signaling, Beverly, MA) containing proteinase inhibitor cocktail (Roche Diagnostics, Indianapolis, IN, USA) to obtain total cellular protein. Protein samples (20-25 *μ*g per lane) were electrophoresed on a 10% or 12% sodium dodecyl sulfate polyacrylamide gel and transferred to polyvinylidene fluoride membranes. After blocking in 5% non-fat milk for 1 h, membranes were incubated with primary antibodies including p-ATM (1:1000), p53 (1:1000), p-p53 (1:1000), p21^Waf1/Cip1^ (p21, 1:1000), caspase-3 (1:1000), cleaved caspase-3 (1:500), and GAPDH (1:2000) (all from Cell Signaling) overnight at 4°C. On the following day, anti-rabbit (for p-ATM, p-p53, p21, caspase-3, cleaved caspase-3 and GAPDH, 1:10000, Santa Cruz, CA, USA) or anti-mouse (for p53, 1:50000, Sigma-Aldrich) peroxidase-conjugated secondary antibodies. Protein bands were detected with chemiluminescent reagents (GE Healthcare, Buckinghamshire, UK) and then exposed to X-ray film.

### 2.6. Immunofluorescent Staining

Cells were seeded on 12 mm coverslips and incubated with corresponding treatments. On the harvest day, cells were fixed with -20°C methanol for 5 min at room temperature. Cells were then incubated with 10% normal goat serum (Dako Corp., Carpinteria, CA) for 1 h, followed by incubation with *α*-tubulin primary antibody (1:200, Santa Cruz) overnight at 4°C. A fluorescein-conjugated secondary antibody (Thermo Fisher Scientific) was used for visualization of the signal. After 1 h of incubation, DAPI counterstaining was performed, and cell counting and the image obtaining were conducted under the fluorescent microscope. The entire procedure was performed under light-proof conditions.

### 2.7. Cell Counting and Statistical Analysis

For cell counting, twenty random fields for each sample were chosen. All positive cells with multiple spinal poles or micronuclei were counted and summed up as the final number for statistical analysis. The number of cells was quantified by using Image J (version 1.50; NIH, Bethesda, MA, USA) in a blinded manner.

All data in the text were expressed as mean ± SD, and statistical analyses were performed using Prism 6 and SPSS 19.0.0. Multiple t-test or one-way ANOVAs with Tukey's multiple comparisons test were performed to evaluate differences among groups. A* p* value < 0.05 was considered statistically significant.

## 3. Results

### 3.1. Caffeine Enhances TMZ's Chemoefficacy in Both Short- and Long-Term Observations

**Figure 2:**
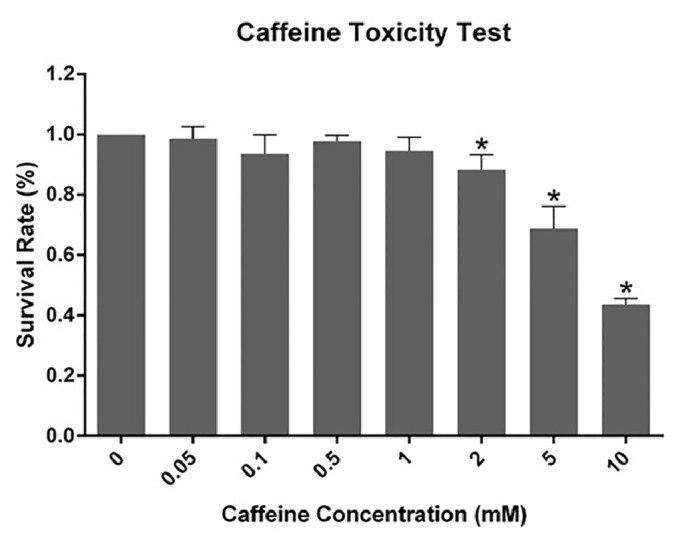
MTT results for caffeine toxicity. Dosages below 1 mM did not affect the viability of U87-MG cells. Therefore, 1 mM was chosen as the target dosage in the further experiments. *∗ p* < 0.05 compared to control.

**Figure 3:**
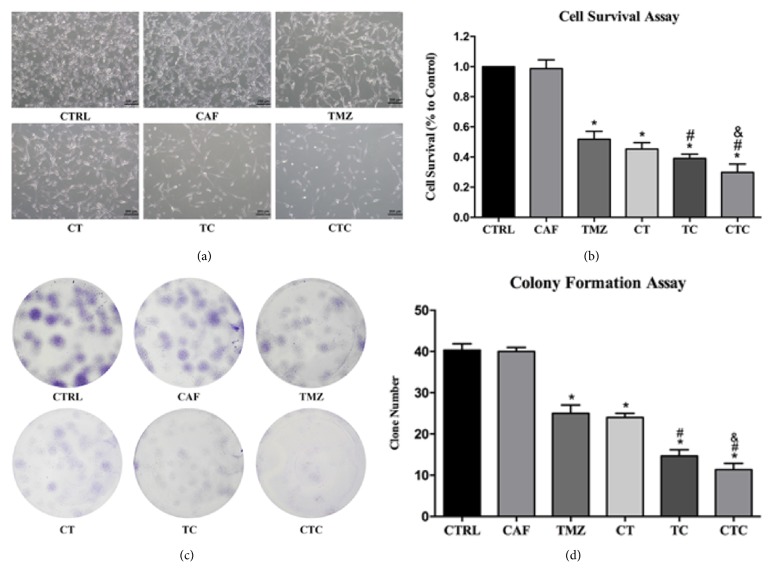
Results of MTT (b) and colony formation assay (c,d). Both assays showed that combination of caffeine and TMZ exerted enhanced antiproliferative effect in comparison with TMZ alone. Furthermore, pretreatment of caffeine produced additional benefits only when it was followed by combined treatment but not TMZ alone. Images from bright field demonstrated the same trend (a). *∗*, #, and & represent for* p* < 0.05 when compared to control, TMZ, and TC, respectively.

As shown in Figure 2, cell viability was not significantly affected by caffeine alone below a dosage of 1 mM during a four-day course; 1 mM was therefore selected for use in subsequent experiments. Three-day incubation with 500 *μ*M TMZ reduced cell proliferation to 50%, with a further 12% reduction after cotreatment with caffeine (TMZ versus TC,* p* < 0.05, Figure 3(b)). Interestingly, pretreatment with caffeine for one day in advance further enhanced the efficacy of cotreatment (TC versus CTC,* p* < 0.05). Though a slightly reduced cell survival was also observed in CT cells (i.e., caffeine followed by TMZ alone), no statistical difference was reached when compared with that of TMZ alone (CT versus TMZ,* p* > 0.05). Microscopic observations concurred with MTT assays and revealed the same trend (Figure 3(a)).

On colony formation assay, 50 *μ*M TMZ alone resulted in an obvious decrease in the number of colonies in comparison with control, and preexposure to caffeine did not bring additional benefits (TMZ versus CT,* p* > 0.05). Concomitant treatment with TMZ and caffeine (i.e., TC group) reduced the number of colonies to 36% of those in CTRL and 58% of those in the TMZ alone group, suggesting an enhanced antiproliferative effect with the use of caffeine. In line with the MTT results, cell growth was further inhibited if cells were given caffeine one day before combined therapy (Figures 3(c) and 3(d)). Altogether, these data suggested that caffeine could enhance TMZ cytotoxicity in both short and long terms.

### 3.2. Caffeine Abrogates TMZ-Induced G2/M Arrest through Inhibiting ATM-p53-p21 Pathway

**Figure 4:**
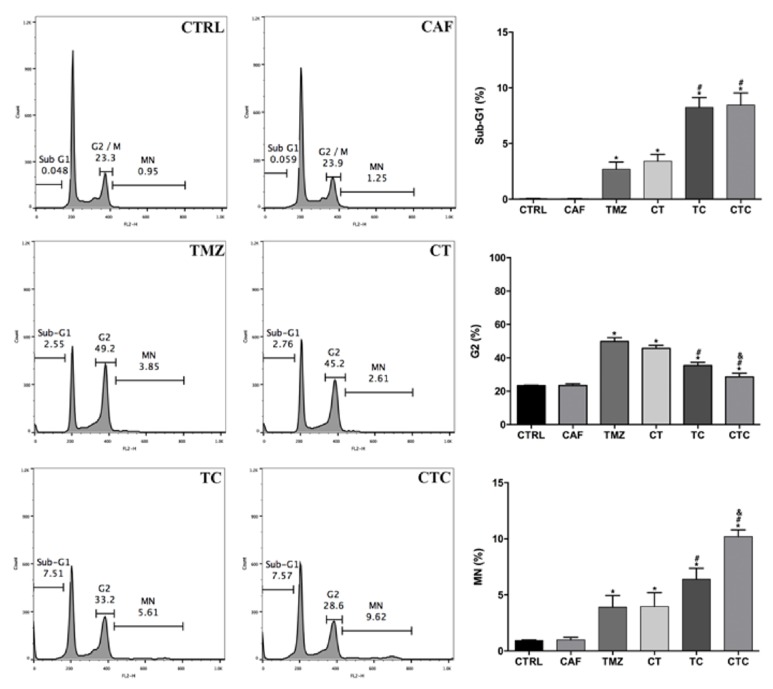
Representative cell cycle analysis in different treatment strategies. Combination of caffeine and TMZ impeded TMZ-induced G2 delay in U87-MG cells and caused an increase in the sub-G1 and MN phase (TMZ versus TC/CTC,* p* < 0.05). An additional increase in MN proportion but not sub-G1 was seen in pretreatment plus combination strategy. *∗*, #, and & represent for* p* < 0.05 when compared to control, TMZ, and TC, respectively.

TMZ causes DNA damage, triggers repair responses, and induces significant cell cycle arrest in glioma cell lines; attenuating this cell cycle delay may facilitate chemocytotoxicity [16]. Caffeine is known as a potent cell cycle modulator by regulating ATM-mediated signaling pathway [17]. Hence, we next asked whether or not this may explain the enhanced chemoefficacy as described above. Flow cytometry results showed that TMZ alone produced a dramatic cycle delay with more than half of the cells being trapped in G2 phase (Figure 4). The presence of caffeine together with TMZ partially abrogated the G2 arrest, as the number of arrested cells decreased by approximately 30% when compared to that observed after TMZ-alone treatment. Meanwhile, the proportion of cells with multinucleation (MN) increased with concomitant caffeine (TMZ versus TC,* p* < 0.05). MN or polyploid peak was usually considered to be a marker of improper cell divisions, characterized by an increased amount of cells containing multiple sets of chromosomes [18]. The obvious increase of MN peak indicated that cotreatment with TMZ and caffeine induced the polyploid formation in U87-MG cells. The results were also in line with the aforementioned findings that the largest amount of cells with MN was seen when caffeine was given both before and during TMZ treatment. Accompanying these results, we also witnessed a sub-G1 peak in four TMZ-treated groups, indicating that TMZ caused apoptotic cell death. Combined treatment again showed a larger amount of cells located in sub-G1 area; however, caffeine pretreatment did not bring additional increase of sub-G1 population, be it followed by TMZ alone or combined treatment (further discussed below).

**Figure 5:**
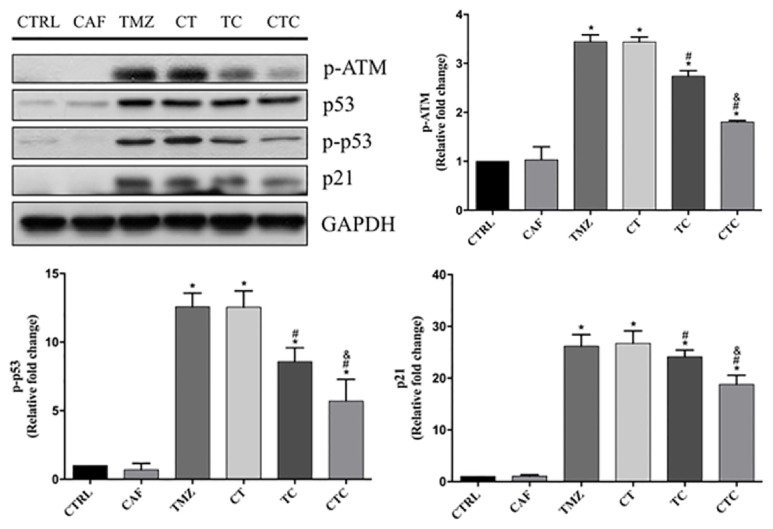
Immunoblotting analysis showed that TMZ-induced activation of ATM/p53/p21 pathway was repressed by concurrent caffeine (TMZ versus TC), and it was further inhibited by preexposure of caffeine 24 h ahead of combined therapy (TC versus CTC). GAPDH was used as an internal loading control. *∗*, #, and & represent for* p* < 0.05 when compared to control, TMZ, and TC, respectively.

Immunoblotting results showed marked ATM phosphorylation in response to TMZ challenge, which concurrently led to an activation of its downstream p53 and p21. Combined treatment with TMZ and caffeine suppressed the activation of ATM/p53/p21 pathway; the phosphorylation of ATM and p53 was restrained, and the expression of p21 was downregulated (TMZ versus TC). Moreover, this effect was further augmented when caffeine was given 24 h ahead of TMZ plus caffeine (TC versus CTC) (Figure 5).

### 3.3. Caffeine Induced Cell Death through Mitotic Catastrophe, Which May Be Independent from Apoptosis

**Figure 6:**
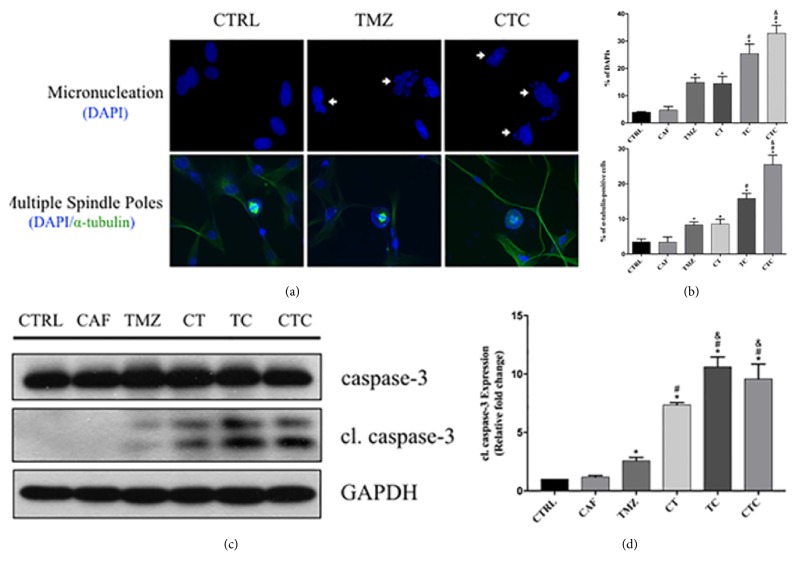
Representative immunofluorescent images illustrating characteristic micronucleation and multiple spindle poles during MC occurrence (a). Proportion of cells showing MC features increased after TMZ treatment, which could be enhanced by concurrent exploitation of caffeine. Pretreatment of caffeine only exerted additional effect when it was followed by combined treatment but not TMZ alone. *∗*, #, and & represent for* p* < 0.05 when compared to control, TMZ, and TC, respectively (b). While combination of caffeine augmented TMZ-induced caspase-3 cleavage, early exposure of caffeine only produced additional benefits to TMZ but not combined treatment. *∗*, #, and & represent for* p* < 0.05 when compared to control, TMZ, and CT, respectively (c, d).

When cells with damaged DNA bypass the cell cycle checkpoint and enter the mitotic phase, they may manifest mitotic catastrophe (MC), which is defined as a mechanism of mitosis-linked cell death due to inappropriate entry into mitosis [19]. In the present study, two characteristic features of MC, micronucleation and mitotic spindle disruptions, were determined. We found that the majority of nuclei were oval-shaped in CTRL cells, while cells with multiple nucleic fragments were observed in TMZ-treated populations (Figure 6(a), DAPI staining). It was more frequently seen in combined treatment (TC) and could be further enhanced when pretreatment was introduced (CTC, Figure 6(b)). Aberrant chromosome segregation represented as multiple spindle poles is also a typical feature of MC. In our studies, over 8% of cells showed multiple spindle poles which indicated mis-segregation of chromosomes in TMZ group; this compared with only around 3% found in CTRL and CAF (Figures 6(a) and 6(b), DAPI and *α*-tubulin counter staining). Then, the number of cells going through multipolar spindle poles doubled when TMZ was combined with caffeine. Consistent with our cell cycle analysis, pretreatment with caffeine only produced additional effect when followed by combined therapy but not TMZ alone, as there were 60% more cells suffering from chromosome mis-segregation in CTC in comparison with TC. These results suggested that combined therapy brought more significant disturbances to chromosome segregation and mitosis that would be indicative of an essential involvement of MC.

We then further investigated the level of cleaved caspase-3, an apoptosis marker. TMZ induced caspase-3 cleavage, which was augmented when cells were preexposed to caffeine (Figures 6(c) and 6(d)). Concurrent treatment then showed a more powerful induction in caspase-dependent apoptosis, while pretreatment did not bring more benefits in this case. These findings were inconsistent with the MC alterations, indicating that caffeine could also work as an apoptotic synergist with TMZ independently from MC. Indeed, apoptosis by sub-G1 (Figure 4) did not show a clear association between apoptosis and MC either, and that no difference was observed between TC and CTC.

## 4. Discussion

Recent epidemiological studies demonstrated the benefits of caffeinated drink intake in decreasing the risk of brain oncogenesis [11, 12]; caffeine alone was also reported to suppress the proliferation and migration of GBM cells both* in vitro* and* in vivo *[13, 14]. Caffeine's synergistic effects with radiotherapy and chemotherapy were also demonstrated in adenocarcinoma cells [20], hepatocellular carcinoma cells [21], and cervical carcinoma cells [22]. In GBM, Janss et al. found that caffeine was a potent sensitizer for cisplatin and camptothecin, as it enhanced the cytotoxicity of both drugs in U251 cells [15]. In line with these findings, our results demonstrated that caffeine at a noncytotoxic concentration promoted the efficacy of TMZ in U87-MG cells. This chemosensitizing benefit produced by concurrent caffeine treatment was likely related to its regulation of cell cycle progressions. It has been known that cell cycle delay upon checkpoint activation following DNA damaging treatments such as TMZ can facilitate DNA repairs, promote cancer cells survival, and lead to chemoresistance [6]. These dormant cells may later exit cell cycle temporarily while remaining metabolically active and are also thought to be more resistant to chemotherapy when they reenter the cell cycle and begin to divide again after a period of time [20]. In the case of GBM, TMZ treatment produces marked cell cycle arrest which could partially explain the high rate of TMZ tolerance and disease relapse in clinical treatment [23]. Our previous works using TMZ-resistant U87-MG and D54-MG lines also revealed dramatic cell cycle arrest after long-term TMZ exposure, indicating that a close association indeed exists between cell cycle delays and the onset of TMZ resistance [24]. Conversely, the attenuation of this cell cycle arrest may potentially promote the efficacy of current chemotherapies and reduce the incidence of chemoresistant relapse.

ATM is one of the essential DNA damage response (DDR) kinases, and its activation can restore genomic integrity in response to DNA instability involved in a variety of cellular processes [25]. Activation of ATM leads to subsequent phosphorylation of downstream substrates, such as p53 and p21, and exerts its effects on DNA repair, cell death, and, most importantly, cell cycle arrest [26]. Therefore, its activation in GBM following TMZ treatment is considered to be responsible for the cell cycle delay due to DNA double strand breaks (DSBs). Pharmacological inhibition of ATM reverses this G2 arrest and renders GBM cells more susceptible to TMZ [27]. In the present study, TMZ dramatically increased the phosphorylation of ATM and its important downstream factors p53 and p21 as anticipated, leading to a substantial G2 stage delay. Caffeine is known as an inhibitor of ATM kinase activity [17], and cotreatment of caffeine suppressed the activation of ATM signaling pathway and ameliorated TMZ-induced G2 arrest, which contributed to the augmentation of TMZ chemoefficacy. These findings are in agreement with previous studies in which caffeine augmented radiotherapeutic benefits by inhibiting ATM and ATR activities in lung adenocarcinoma cells and leukemia cells [28].

We also observed an inhibition of phosphorylation of p53, a well-known tumor suppressor, through the suppression of ATM activation in combined TMZ plus caffeine treatment. Extensive studies had shown correlations between p53 functional loss and tumorigenesis of liver cancer, lung cancer, colon cancer, and GBM [29]. Activation of p53 plays a crucial role in TMZ therapies in GBM treatment as it is believed to mediate apoptotic cell death following ATM-mediated DDR [30]. In this regard, one might expect that downregulating p53 would work against DNA damaging agents in the treatment of GBM. However, current evidence suggests that p53 participation in anticancer therapy exhibits a two-armed effect; in addition to apoptosis, p53 mediates checkpoint activation and induces cell cycle delay which is considered to favor DNA repair and attenuate chemoefficacy [31]. In the present study, although the phosphorylation of p53 was significantly suppressed, chemoefficacy was augmented as both mitotic cell death and apoptosis were enhanced following treatment with both caffeine and TMZ (TMZ versus TC). This result agreed with several works which demonstrated that GBM cells with inactive p53 were more sensitive to TMZ treatment than those with wild-type p53 [32, 33].

We surmised, and our findings suggested, that GBM cells that reenter cell cycle following our treatments would carry impaired DNA and experience aberrant and incomplete mitosis. The latter may lead to cell division failure and cellular break down. This form of catastrophic cell division, or MC, is characterized by the distinguishing features of giant cells with micronuclei formation and multiple spindle poles, both of which reflect an abnormal segregation of chromosomes [34]. G2 arrest prevents MC by halting premature entry, and MC will manifest when G2 arrest is abrogated. In this work, we observed an inverse association between the extent of G2 arrest and frequency of MC. The number of cells showing large cell bodies with micronucleation and multipolar spindles was larger in combined treatment groups when compared with TMZ alone. This trend was in line with the alterations in cell cycle analysis where G2 peak was lower and MN peak was higher in TC and CTC groups in comparison with TMZ alone. Our results supported the notion that caffeine produced its chemosensitizing effects by promoting mitotic cell death through abrogating TMZ-induced G2 delay.

So far, there is no general consensus on the relationship between MC and apoptosis [35]. Caspases were reported to be essential for the termination of MC, indicating that premature mitotic failure might act as an intermediate process leading to ultimate apoptotic cell death [36, 37]. On the other hand, in p53-deficient U-2 OS bone osteosarcoma cells, MC could be induced by adeno-associated virus in the absence of caspase activation and apoptosis [38]. In another MDR1-induction model in HeLa cells, apoptosis was significantly suppressed, while cellular fractions resulted from MC increased after ionizing radiation [39]. The obvious phenotypic differences between apoptosis and MC also supported the notion that these two cellular processes may be independent. Herein, although combined treatment enhanced both apoptosis and MC, early caffeine exposure only brought additional benefits to apoptotic cell death when followed by TMZ alone, which was in contrary with its effect on MC development. Supportively, a previous study using SH-SY5Y human neuroblastoma cell line showed that pretreatment with caffeine increased the sensitivity of cells to doxorubicin-induced apoptosis secondary to an increased production of mitochondrial-free radical [40]. Together with our data, we propose that caffeine pretreatment before the administration of chemotherapeutics could sensitize glioma cells to apoptosis that is likely independent from MC.

Last but not least, it was interesting to observe that caffeine-pretreated cells showed an enhanced sensitivity to combined treatment through MC augmentation but not to TMZ alone. It has been reported that long and chronic caffeine exposure would sensitize organisms to some sort of drugs such as methylphenidate [41], but the mechanism is far from clear. In the study by Susan et al., although the caffeine-induced sensitization of neuroblastoma cells to doxorubicin was attributed to increased mitochondrial-free radical production, a decrease of total reactive oxygen species (ROS) production was also noticed [40]. Evasion of ROS production by antioxidants was reported to facilitate MC development [42], indicative of a potential involvement of ROS regulation in a setting similar to our study. Note should be taken that potentiation by caffeine to chemotherapy is cell line- and agent-specific [40]; therefore, further studies in other GBM cell lines as well as in animal models are needed.

## 5. Conclusions

**Figure 7:**
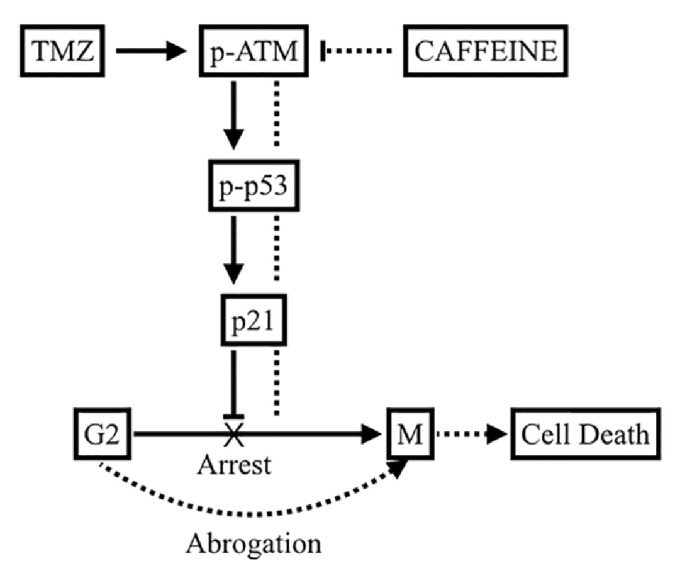
Proposed mechanisms for caffeine in sensitizing TMZ's efficacy. TMZ causes DNA damage, activates ATM/p53/p21 pathway, and induces significant G2 arrest (full line). Caffeine inhibits the phosphorylation of ATM and thus suppresses the activation of DDR pathway. G2 delay is abrogated, cells with damaged DNA enter mitotic phase prematurely, and augmented MC occurs (dotted line). Our results did not show a dependent association between MC and apoptosis; hence further studies are warranted.

The present study demonstrated that caffeine enhanced TMZ's chemoefficacy through impeding G2 delay by inhibiting ATM/p53/p21 pathway and the promotion of mitotic catastrophe (Figure 7). Our results highlighted the following. (1) Caution should be paid to the fact that cancer cells possess different intrinsic responses to chemotherapies, and GBM is known to be resistant to apoptosis. TMZ induces significant cell cycle arrest at an early stage of treatment rather than cell death, which could account for the frequent clinical relapses seen after TMZ treatment. (2) Attenuating G2 delay after TMZ treatment enhances MC, which may then lead to cellular breakdown in ways that may be either dependent or independent from apoptosis. This particular property should be further explored in the treatment of GBM. (3) Caffeine pretreatment augments the sensitizing efficacy of combined treatment, indicating the potential benefit of a high intake of caffeinated products before and during TMZ treatment.* In vivo* confirmation of our findings as well as epidemiological studies is needed.

## Data Availability

The data used to support the findings of this study are available from the corresponding author upon request.
